# Exocytosis of Varicella-Zoster Virus Virions Involves a Convergence of Endosomal and Autophagy Pathways

**DOI:** 10.1128/JVI.00915-16

**Published:** 2016-09-12

**Authors:** Erin M. Buckingham, Keith W. Jarosinski, Wallen Jackson, John E. Carpenter, Charles Grose

**Affiliations:** Departments of Pediatrics and Microbiology, University of Iowa, Iowa City, Iowa, USA; Northwestern University

## Abstract

Varicella-zoster virus (VZV) is an extremely cell-associated herpesvirus with limited egress of viral particles. The induction of autophagy in VZV-infected monolayers is easily detectable; inhibition of autophagy leads to decreased VZV glycoprotein biosynthesis and diminished viral titers. To explain how autophagic flux could exert a proviral effect on the VZV infectious cycle, we postulated that the VZV exocytosis pathway following secondary envelopment may converge with the autophagy pathway. This hypothesis depended on known similarities between VZV gE and autophagy-related (Atg) Atg9/Atg16L1 trafficking pathways. Investigations were carried out with highly purified fractions of VZV virions. When the virion fraction was tested for the presence of autophagy and endosomal proteins, microtubule-associated protein 1 light chain (MAP1LC3B) and Ras-like GTPase 11 (Rab11) were detected. By two-dimensional (2D) and 3D imaging after immunolabeling, both proteins also colocalized with VZV gE in a proportion of cytoplasmic vesicles. When purified VZV virions were enumerated after immunoelectron microscopy, gold beads were detected on viruses following incubation with antibodies to VZV gE (∼100%), Rab11 (50%), and LC3B (30%). Examination of numerous electron micrographs demonstrated that enveloped virions were housed in single-membraned vesicles; viral particles were not observed in autophagosomes. Taken together, our data suggested that some viral particles after secondary envelopment accumulated in a heterogeneous population of single-membraned vesicular compartments, which were decorated with components from both the endocytic pathway (Rab11) and the autophagy pathway (LC3B). The latter cytoplasmic viral vesicles resembled an amphisome.

**IMPORTANCE** VZV infection leads to increased autophagic flux, while inhibition of autophagy leads to a marked reduction in virus spread. In this investigation of the proviral role of autophagy, we found evidence for an intersection of viral exocytosis and autophagy pathways. Specifically, both LC3-II and Rab11 proteins copurified with some infectious VZV particles. The results suggested that a subpopulation of VZV particles were carried to the cell surface in single-walled vesicles with attributes of an amphisome, an organelle formed from the fusion of an endosome and an autophagosome. Our results also addressed the interpretation of autophagy/xenophagy results with mutated herpes simplex virus lacking its ICP34.5 neurovirulence gene (HSVΔ34.5). The VZV genome lacks an ICP34.5 ortholog, yet we found no evidence of VZV particles housed in a double-membraned autophagosome. In other words, xenophagy, a degradative process documented after infection with HSVΔ34.5, was not observed in VZV-infected cells.

## INTRODUCTION

Macroautophagy, here referred to as autophagy, is an ancient survival mechanism (1). Similarly, herpesviruses are ancient viruses; for example, the herpesvirus of oysters is presumed to have coevolved with mollusks, contemporary marine invertebrates originating during the Cambrian period over 500 million years ago (mya). Autophagy is upregulated during infection with oyster herpesviruses (2).

Autophagy is also upregulated during infection with varicella-zoster virus (VZV), human herpesvirus 3, another herpesvirus of a more recent lineage in geologic time, perhaps 70 mya (3). The VZV genome (124 kbp) is the smallest among the human herpesviruses (4). VZV is unusual in that it causes two distinguishable diseases in humans: varicella after primary infection in childhood and herpes zoster after reactivation later in life (5). Each disease is associated with a vesicular rash (exanthem), sites of virus replication in the skin. The cells at the base of the vesicles are filled with autophagosomes (6). This induction of autophagy following VZV infection is easily reproduced after infection of either cultured cells or xenografts in the severe combined immunodeficient mouse model of VZV pathogenesis (7–9). Furthermore, inhibition of autophagy decreases the infectivity titer; thus, autophagy is proviral (10, 11).

Among the herpesviruses, VZV is renowned for its extreme cell association with impaired egress of viral particles and low infectivity; the particle-to-PFU ratio is 40,000:1 (12–14). Upon exocytosis, most viral particles appear to remain attached to the outer cell membrane and are never released into the overlying culture medium (15, 16). Furthermore, there are virtually no data about the vesicle that transports the viral particles from the virus assembly compartment to the outer cell membrane (17, 18). Based on the proviral aspects of autophagy that we recently observed in VZV-infected cells, we postulated a role for autophagy during this portion of the VZV life cycle. To this end, we have examined whether there is evidence for a convergence of viral exocytosis and autophagy pathways following VZV secondary envelopment. As part of this project, we also reexamined numerous electron micrographs to clarify the membrane structure of the vesicle that harbors VZV virions after secondary envelopment. The results support a previously unrecognized role for an amphisome or a closely related single-membraned vesicle as an exocytosis compartment for some of the egressing virions (19).

## MATERIALS AND METHODS

### Virus strains and cells.

VZV strains included the low-passage-number laboratory strain VZV-32 and the attenuated VZV Oka strain (vOka); both strains have been sequenced (20, 21). Each of the VZV open reading frames (ORFs) within the DNA genome has been designated sequentially (left to right) by the numbers 0 to 71 (GenBank accession number NC_001348) (4). The main VZV protein discussed in this paper is the product of ORF68 (also called gE); in previous reports, the VZV gE protein was called gp98 or gpI (22). The virus was passaged in monolayers of either human diploid fibroblasts (strain MRC-5) or human melanoma cells (strain MeWo) as described previously (23). The technique for the production of cell-free virus by sonication of infected monolayers was described in detail previously (24).

### Antibody reagents and immunoblotting.

Antibody reagents included anti-VZV murine monoclonal antibodies (MAbs) 3B3 (anti-ORF68; gE), 5C6 (anti-ORF62), and 251D9 (anti-ORF41), produced in our laboratory (25); anti-VZV gE guinea pig antibody, produced in our laboratory; anti-gE MAb (catalog number sc-56995; Santa Cruz); anti-LC3B antibody (rabbit, catalog number L7543 [Sigma] or catalog number sc-28266 [Santa Cruz]); anti-LC3A antibody (rabbit, catalog number 11668-019AP-1802a; Abgent); anti-Ras-like GTPase 11 (Rab11) MAb (mouse, catalog number sc-166912; Santa Cruz); antireovirus murine MAb as an IgG2a isotype antibody control (catalog number H4A3; NIH Developmental Studies Hybridoma Bank); and anti-lactate dehydrogenase (LDH) MAb (catalog number sc-133123; Santa Cruz). In particular, MAb 3B3 (isotype IgG2a) against a defined epitope on VZV gE is one of the most highly characterized VZV-specific antibody reagents, with known kinetic and equilibrium constants (20). Hoechst H33342 dye was added for nucleus staining (catalog number H3570; Life Technologies). Fluoroprobes included as secondary antibodies included goat anti-mouse Alexa Fluor 633, goat anti-rabbit Alexa Fluor 488, goat anti-guinea pig Alexa Fluor 546, goat anti-mouse Alexa Fluor 546, goat anti-mouse IgG1 Alexa Fluor 568, and goat anti-mouse IgG2a Alexa Fluor 633 (catalog numbers A11017, A11018, A11070, A11074, A21053, A21124, and A21136; Life Technologies). Immunoblotting techniques were described previously by our laboratory (10).

### Protocol for purification of VZV virions.

Our laboratory reported several studies between 1979 and 1983 that detailed methodology for VZV virion purification and enumeration of the structural and nonstructural viral proteins (26–29). Initial experiments were modeled after the strategy reported previously by Spear and Roizman to enumerate the herpes simplex virus (HSV) infected cell-specific (ICS) proteins (30). However, we quickly discovered that VZV is highly cell associated with no released virus; therefore, we could not repeat the precise ladder of ICS proteins seen in released HSV. To circumvent this obstacle, we first made a highly specific polyclonal anti-VZV antibody followed by several panels of murine monoclonal antibodies (25). With these antibody reagents, we demarcated a ladder of specific VZV-precipitable proteins and glycoproteins. The accuracy of our analyses was verified when the complete genomic sequence of VZV was reported in 1986 (31). To distinguish structural from nonstructural products, we delineated methods of virion purification by density gradient sedimentation. Again, compared with released HSV-1, VZV virions within cell-free virus prepared from sonicated infected monolayers were considerably more fragile, with a frequent loss of the outer envelope during any high-speed centrifugation (32). After examination of several different media, we settled upon a density-viscosity gradient of potassium tartrate (KT) and glycerol as the preferred medium for the purification of VZV virions that retained some infectivity.

For the above-mentioned reasons, cell-free virus required for the studies outlined below was harvested from a 150-cm^2^ infected MRC-5 cell monolayer and layered in a 1-ml aliquot onto a preformed KT-glycerol gradient. The gradient was prepared by mixing 7 ml of 50% (wt/vol) KT and 8 ml of 30% (vol/vol) glycerol in a dual-chambered gradient maker feeding into a 16- by 102-mm centrifuge tube (catalog number 344061; Beckman Coulter). The gradient was sedimented for 16 h at 115,000 × *g* in a Beckman SW27 rotor at 4°C. After the first sedimentation, there was an obvious particular band (infectivity virus band) in the lower one-third of the gradient and a less apparent opalescent band (light particle band) in the upper one-third. Each band was removed and placed onto a second potassium tartrate-glycerol gradient for a second sedimentation under identical conditions. After the second sedimentation, the lower and upper bands were removed and diluted, and their contents were concentrated by a subsequent sedimentation for 1 h at 124,000 × *g*.

### Confocal laser scanning microscopy with two-dimensional (2D) and 3D imaging.

Monolayers grown on glass coverslips were labeled with antibodies and fluorophores as described above, mounted onto glass slides, and then viewed with a Zeiss LSM710 confocal laser scanning microscope, using 10× and 20× dry objective lenses and 40× and 63× high-numerical-aperture oil immersion objective lenses (33, 34). The image size was set to either 512 by 512 or 1,024 by 1,024 pixels. Emission detection bandwidths were configured by Zeiss Zen control software. Both three-dimensional (3D) renderings and animations of confocal data sets were created with Imaris software version 8.0 (Bitplane), and subsequent analyses were carried out with its built-in tools (34). In particular, we used the Imaris spot detection algorithm and colocalization analysis (35).

### Quantitation of colocalization.

Confocal images (z-stacks acquired by using the 63× objective) from each experiment described below in Results were analyzed by using the Cell Counter plug-in for ImageJ (36). At least 50 cells were analyzed for each set of staining conditions, based on H33342 DNA staining to identify nuclei. Only cells with complete nuclei in individual optical sections were counted. Colocalization was quantitated as follows: percent colocalization = [(number of yellow puncta)/(number of total puncta containing red)] × 100. Multiple images per condition were analyzed, totaling at least 50 cells per condition, and standard deviation (SD) was the measure selected to generate error bars.

### Electron microscopy of purified virus fractions.

Pelleted viral fractions from the KT-glycerol gradients were resuspended in 200 μl of phosphate-buffered saline (PBS) after a short (5-s) sonication burst. Next, 25 μl of this material was dropped onto carbon-coated transmission electron microscopy (TEM) grids (Formvar-coated nickel TEM grids that were carbon coated) and incubated at room temperature (RT) for 1 h, with occasional mixing by movement of the grids in the drop. Washing of TEM grids was done by briefly dipping the grids into water and then suspending the grids on drops of water (25 μl). After adsorption of viral material onto the grids, the grids were washed and then blocked in 5% nonfat milk with 5% normal goat serum, and the grids were incubated in drops of primary antibody (1:50 dilution) at RT for 4 h and then overnight at 4°C. After washing, the grids were incubated with secondary immunogold antibody (goat anti-mouse or goat anti-rabbit ultrasmall gold beads; 1:25 dilution) for 4 h at RT. After washing, the grids were suspended in freshly prepared silver enhancement solution (Aurion) for 30 min, washed with water, and then negatively stained with 2.5% uranium acetate drops for 2 min at RT. The uranyl acetate was wicked off with filter paper, and the grids were allowed to air dry overnight. Grids were viewed with a JEOL 1230 transmission electron microscope.

### Cryo-electron microscopy (cryo-EM).

Cells were scraped into minimal essential medium (MEM) containing 1% gelatin with a rubber policeman. In a series of centrifugation and medium replacement steps, the cells were fixed with 2% paraformaldehyde and 0.5% glutaraldehyde in PBS for 30 min at RT, followed by increasing concentrations of warm gelatin (4%, 6%, 8%, 10%, and 12%) in PBS. The final gelatin solution was allowed to solidify at RT and then placed at 4°C overnight. The solid gelatinized cells were incubated in a 2 M solution of sucrose in PBS at RT overnight. The resulting solid sample was plunge frozen in liquid nitrogen, and thin sections were cut with a cryoultramicrotome and collected on grids. The resulting sections were then antibody labeled as described above, poststained with 2.5% uranium acetate and 2% lead citrate for 5 min, and then washed and dried before viewing with a JEOL 1230 transmission electron microscope.

## RESULTS

### Colocalization of VZV gE with autophagy proteins during VZV infection.

Our previous studies of autophagy documented the important observation that autophagic flux is not inhibited by VZV infection. In fact, the converse is true; namely, inhibition of flux inhibits virus spread and viral titers. Based on these observations, we postulated that components of the autophagy pathway were contributing to the VZV infectious cycle. Also, based on extensive previous imaging studies, we postulated that any intersection of the autophagy and endosomal pathways would be detectable in VZV-infected cells by probing the pathways for a major marker of the autophagy pathway, microtubule-associated protein 1 light chain 3B (MAP1LC3B or simply LC3B). Confocal laser scanning microscopy is an ideal technology for these investigations because its resolution and optical sectioning capacities facilitate the visualization of structures with a diameter of 400 to 1,000 nm, such as an autophagosome (34).

**FIG 1:**
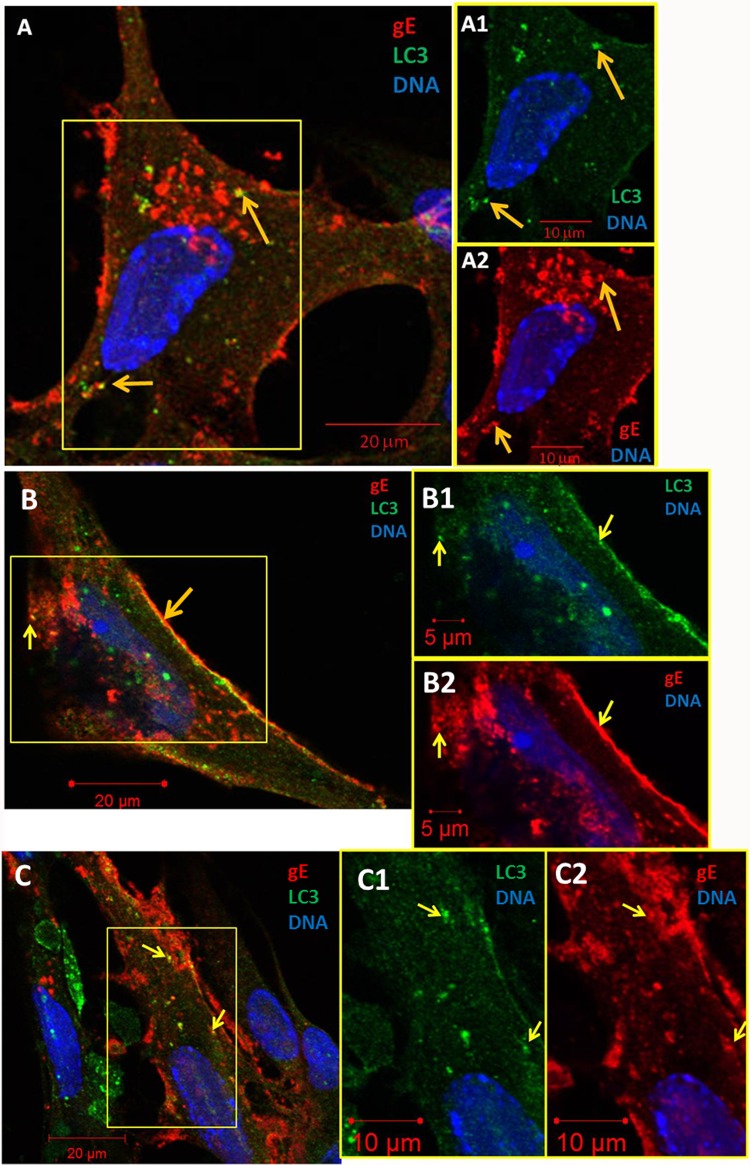
Colocalization of VZV gE with the autophagy protein LC3B. At 72 hpi, MRC-5 monolayers were fixed with 2% paraformaldehyde and then processed for examination by confocal microscopy as described in Materials and Methods. Monolayers were incubated with rabbit anti-LC3B primary antibodies, followed by murine MAb anti-gE. (A) Representative image (magnification, ×630). The boxed area is split into separate channels in panels A1 and A2. Yellow arrows point to representative gE puncta that colocalized with LC3B. Two puncta are in the top panel, while another is in the bottom panel. Not all puncta with colocalization are arrowed. (B) Representative image. Yellow arrows show gE/LC3B colocalization in the cytoplasm and at the edge of an infected cell in both merged and split images (B1 and B2). (C) Representative image. Yellow arrows also point out two colocalized puncta near the cell membrane in both merged and split images (C1 and C2).

**FIG 2:**
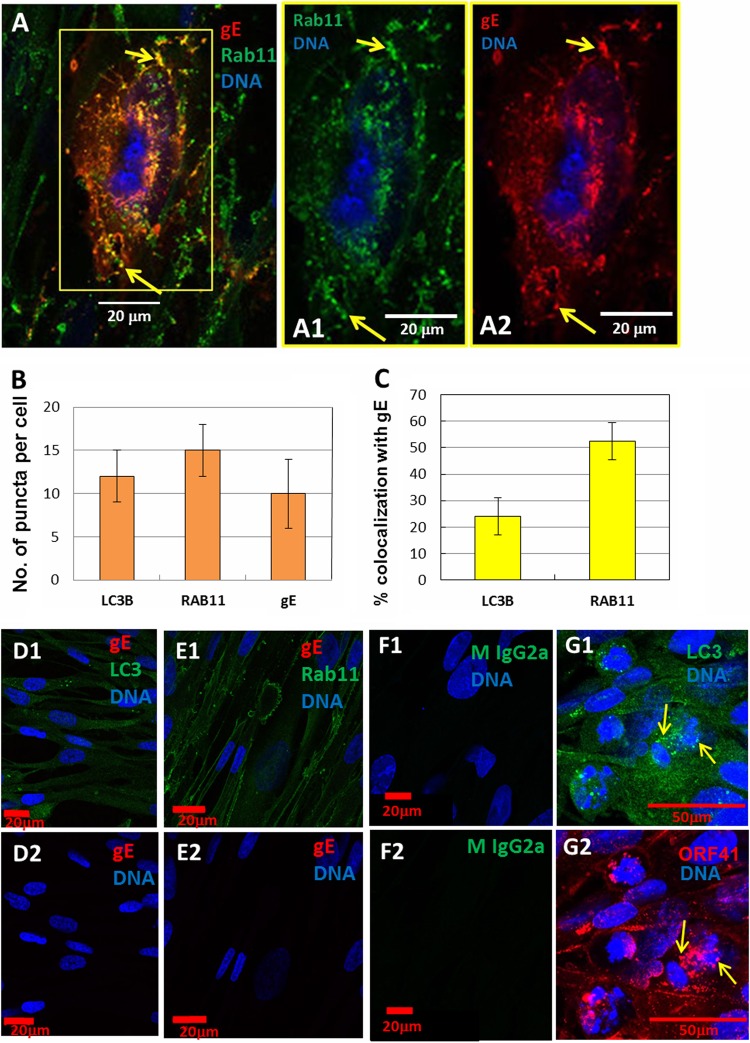
Colocalization of the VZV gE protein with Rab11 protein and control experiments for immunolabeling. Incubations with primary antibody were performed sequentially. First, antibody to Rab11 was added to cells. Cells were then washed and incubated with MAb 3B3 (anti-gE MAb). (A) Representative image of a VZV-infected cell. Yellow arrowheads indicate two puncta with colocalization between gE and Rab11. Not all puncta with colocalization are arrowed. The boxed area is split into separate channels (A1 and A2). (B) Quantitation of numbers of LC3B, Rab11, and gE puncta per cell. (C) Calculation of percentages of gE puncta per cell that colocalized with either the LC3B or Rab11 protein. (D1) Uninfected monolayer with antibodies to gE and LC3B plus DNA stain. (D2) Uninfected monolayer with antibody to gE plus DNA stain. (E1) Uninfected monolayer with antibodies to gE and Rab11 plus DNA stain. (E2) Uninfected monolayer with antibody to Rab11 plus DNA stain. (F1) Infected monolayer with an isotype antibody control plus DNA stain. (F2) Infected monolayer with an isotype antibody control without DNA stain. (G1) Infected monolayer immunolabeled with antibodies to ORF41p and LC3B. Colocalization is indicated by arrows. The merged image is not shown. This image includes channels for LC3B and nuclei. (G2) Image showing channels for ORF41p and nuclei.

We had not observed colocalization in previous autophagy studies when we examined cultures labeled with individual antibody fluoroprobes to LC3B and VZV gE. We proposed that our high-affinity murine anti-VZV gE MAb 3B3 may have obscured binding by a lower-affinity polyclonal anti-LC3B antibody in previous experiments. For the present experiments, we added the anti-LC3B antibody before the addition of the high-affinity anti-gE antibody. Using this strategy, we observed some colocalization between LC3B and VZV gE in cytoplasmic vesicles (Fig. 1). Because of the importance of this observation, we selected three separate representative confocal images, each including a merged image and two split images (Fig. 1A to C). Some puncta with colocalization were most apparent near the edge of the outer membrane of the infected cell (Fig. 1B). Additional quantitation data and control experiments are shown in Fig. 2.

### Colocalization of VZV gE with endosomal proteins during VZV infection.

Based on positive LC3B results, we expanded our colocalization studies to include the enumeration of gE puncta with Rab11 protein, which is found on recycling endosomes. To this end, VZV-infected MRC-5 monolayers were first probed with an antibody to Rab11, followed by an antibody to the VZV gE protein. By 2D confocal microscopy, there was obvious colocalization of some VZV gE with Rab11 (Fig. 2A). Quantitation is included in Fig. 2B and C. There were more Rab11-positive (Rab11^+^) puncta per cell than LC3B^+^ puncta per cell (Fig. 2B), and there was more Rab11/gE colocalization (53%) than LC3B/gE colocalization (24%) (Fig. 2C). Note that the average number of LC3B^+^ puncta per cell in Fig. 2 was calculated differently from a previous report in which we subdivided infected cells by their phase within the VZV infectious cycle; thus, in that previous report, some infected cells had a higher number of LC3B^+^ puncta. The number of puncta in an unstressed and uninfected cell is <4.

Several additional control experiments were performed; these included the incubation of gE and LC3B antibodies in uninfected cells (Fig. 2D), gE and Rab11 antibodies in uninfected cells (Fig. 2E), and an antireovirus isotype control antibody in infected cells (Fig. 2F). All three sets of control experiments showed no nonspecificity. We also performed another control experiment on infected cells, using a different anti-gE MAb that was of the IgG1 isotype and the same antireovirus MAb (isotype IgG2a); again, there was no nonspecificity (data not shown). Since all previous studies were performed with antibody probes to detect the major VZV structural glycoprotein gE, we also carried out a control experiment to detect colocalization with a nonglycosylated VZV capsid protein. Although the nonglycosylated VZV structural proteins have not been as well characterized as gE, we have a MAb reagent against the capsid ORF41p (HSV UL18 ortholog) (37). We repeated a colocalization experiment with this reagent and found ORF41p in LC3-positive cytoplasmic compartments (Fig. 2G). The appearance of these puncta was similar to the appearance of those with gE/LC3 colocalization (Fig. 1). Because the vast majority of VZV trafficking studies are based on gE, subsequent studies will concentrate on pathways outlined by this well-characterized viral product (38).

In order to expand the 2D results and observe 3D colocalization, we rendered the large data sets of z-stack images in the Imaris software program in order to use the spot detection algorithm and colocalization analysis. The Imaris software program facilitated a clearer examination of the microrelationship between nuclei and puncta within a cell, for example, to detect the orientation of puncta labeled for both Rab11 and gE. In particular, spots were easily seen above the nuclei extending toward the outer cell membrane (see Video S1 in the supplemental material). Near the outer cell membrane, the spots are oriented in linear rows called viral highways in a previous report (16).

### Purification of infectious virus and light particles.

**FIG 3:**
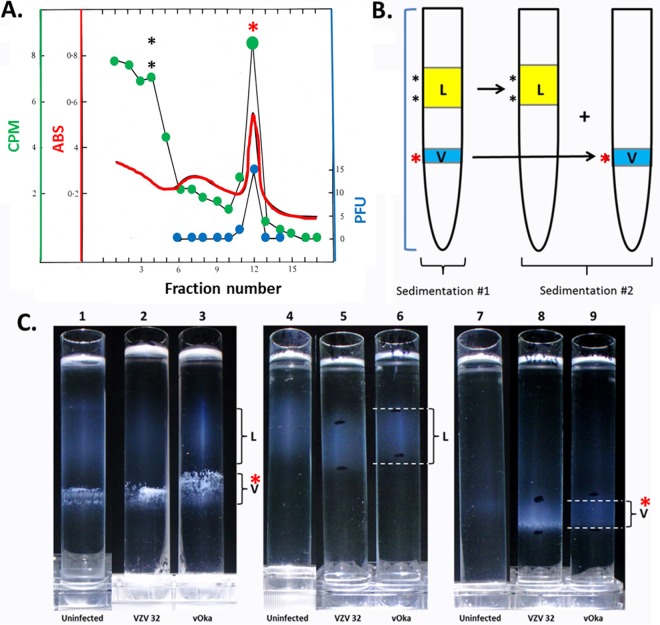
Purification of infectious VZV particles from infected monolayers. (A) Graph of the fractionation of typical density-viscosity gradient centrifugation. The graph delineates that the sole fraction, from the first gradient purification step, with virus infectivity was found in fraction 12. The same fraction had a peak of radioactivity of ^14^C-labeled amino acids. The even-numbered fractions were examined by electron microscopy; only fraction 12 contained enveloped capsids. ABS, absorbance. (B) Diagram of sedimentations using the VZV-32 laboratory strain and the vaccine strain vOka. The sedimentations were carried out twice. The first gradient contains both a virus fraction (V) and a light (L) particle fraction. Each fraction was subsequently sedimented through a second density-viscosity gradient to further decrease contamination by cellular organelles. (C) Photographs of the first (lanes 1 to 3) and second (lanes 4 to 9) sedimentations to illustrate differences between and locations of the virus and L-particle fractions. Red asterisks in panels A to C mark corresponding infectivity fractions/bands.

In order to determine if gE/LC3B and gE/Rab11 colocalization events were occurring in cytoplasmic vesicles containing virions, we required purified virus. VZV virions are very difficult to analyze, because this cell-associated virus is not released into the culture medium overlying infected monolayers. Therefore, to obtain infectious virus free of cellular components, viral particles must be isolated by equilibrium ultracentrifugation (Fig. 3A) (26, 27). First, infected cell monolayers (150 cm^2^) were harvested and then subjected to sonication. Two viral strains were included: the wild-type VZV-32 strain and the attenuated VZV Oka vaccine strain. An uninfected monolayer was subjected to the same experimental conditions. After removal of cellular debris from a sonicated monolayer by low-speed sedimentation, infectious virus was purified from the supernatant by an ultracentrifugation protocol: density-viscosity gradients made from potassium tartrate and glycerol. After a 16-h high-speed sedimentation, the virus-enriched band (V) (infectivity fraction) and the light particle band (L) (fraction with noninfectious enveloped particles lacking a capsid) were isolated from the first density gradient, and each band was then layered onto a separate second density gradient for a second high-speed sedimentation in an ultracentrifuge (Fig. 3B). Uninfected cells were similarly processed. Because of the difficulty in purifying infectious VZV virions and because there is no established protocol by which to purify vOka, we included photographs of the centrifugation gradients at each step of the process.

As shown in Fig. 3C, there was an obvious particulate (lower) band containing infectious virus in all purifications from infected cells. This intensity of the virus band (V) decreased after the second sedimentation (Fig. 3C, compare lanes 2 and 3 with lanes 8 and 9). The opalescence of a more diffuse upper band containing light particles (L) increased modestly over the two sedimentations (Fig. 3C, compare lanes 2 and 3 with lanes 5 and 6). Of importance, the particulate band sedimenting at the same density/viscosity as the infectious virus band in the uninfected cell experiment all but disappeared between the first and second ultracentrifugations (Fig. 3C, compare lanes 1 and 7). These results indicated that the infectious particles themselves were largely responsible for the particulate virus band seen in the second sedimentation. We previously observed each of the even-numbered density gradient fractions shown in Fig. 3A by electron microscopy; only fraction 12 (V) contained enveloped capsids (26, 27).

### Detection of autophagy and endosomal proteins in fractions with virus and light particles.

In order to verify that the infectious virus fraction and the light particle fraction in the second sedimentations contained viral products, aliquots were solubilized and probed for the predominant VZV structural glycoprotein gE. VZV gE has been the standard marker for VZV virions since the earliest studies of VZV purification methods, because the protein is easily detectable by Western blotting with MAb 3B3, which binds to a defined linear epitope (amino acids 150 to 162) on the N terminus (20, 38).

**FIG 4:**
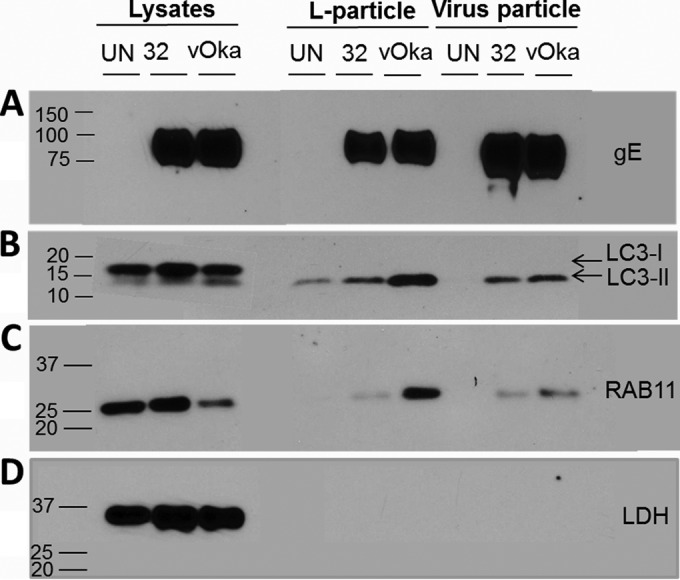
Detection of VZV gE, LC3-II, and Rab11 proteins in the purified VZV and L-particle fractions. As described in the legend of Fig. 3, VZV-32- and vOka-infected monolayers as well as an uninfected control monolayer (UN) were sonicated and subjected to two density gradient sedimentations. The upper L-particle and lower infectious virus bands were isolated, pelleted, and then resuspended in 200 μl of SDS sample loading buffer. Aliquots of identical volumes (20 μl) from these samples were then electrophoresed on gradient SDS-PAGE gels, transferred onto polyvinylidene difluoride membranes, and Western blotted for VZV gE (A), LC3B (B), Rab11 (C), and LDH (loading control) (D) proteins. Molecular weight markers are included in the left margins of each panel.

As shown in Fig. 4A, the gE protein was easily detected in both the lower and upper bands for both strains (VZV-32 and vOka). This result confirmed sequencing data that showed an intact epitope in vOka gE. We previously verified the nature of these two bands with an antibody against the VZV capsid ORF41p; namely, the lower virus band was positive for ORF41p, while the same protein was absent from the upper L-particle band (37).

To further pursue our hypothesis about a convergence of the viral secondary envelopment-and-trafficking pathway with the autophagy pathway, we investigated whether the lipidated form of the LC3 protein (LC3-II) was detectable in either the purified virus fraction or the light particle fraction after the second sedimentation (Fig. 4B). When the virus and light particle bands were solubilized, they were subjected to electrophoresis, transferred onto a membrane, and probed with a rabbit anti-LC3 antibody. LC3-II protein was detected in virus and L-particle bands of both virus strains. We observed slightly less LC3-II protein in the wild-type VZV-32 fractions than in the vaccine strain vOka, especially in the L-particle bands. The LC3-I band was not visible at this exposure in the vOka lane but was visible after a longer exposure (data not shown). In the control samples taken from uninfected and infected cell lysates before any centrifugation, LC3-I and LC3-II proteins were detected. As expected, the bands in the uninfected culture did not contain the VZV gE protein (Fig. 4B, lane UN).

Furthermore, we also probed for the Rab11 protein, based on the data from confocal microscopy (Fig. 4C) (39, 40). The immunoblot for Rab11 was positive in both the virus and L-particle bands for both viruses, although more protein was seen in the vOka lanes. Finally, we also analyzed these samples for the presence of a cellular protein that was predicted not to be a part of the viral particle or vesicle. Lactate dehydrogenase (LDH) provided a negative control and also served as a loading control in the lysate lanes. When fraction samples were immunoblotted with an anti-LDH antibody, no reactivity was observed in the virus and L-particle lanes (Fig. 4D). This result provided evidence that the association between gE and autophagy pathway proteins was not due to nonspecific binding.

### Examination of VZV particles by immunoelectron microscopy.

**FIG 5:**
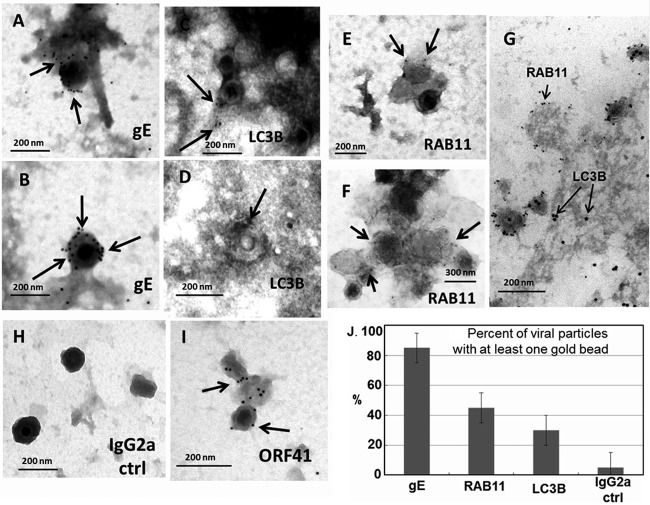
Immunodetection of VZV gE, LC3, and Rab11 proteins on VZV particles. VZV-32-infected monolayers were sonicated and sedimented by equilibrium centrifugation twice (Fig. 3). Aliquots of the lower virus band were placed onto TEM grids. After processing, the grids were immunolabeled with antibody to VZV gE (MAb 3B3), the VZV ORF41 protein (MAb 251D9), LC3B, or Rab11, followed by a secondary antibody conjugated to ultrasmall gold beads or 10-nm gold beads. (A and B) Representative viral particles that exhibited gold beads labeling VZV gE in the envelope (arrows). (C and D) Viral particles that exhibited gold beads labeling LC3B-II in the envelope (arrows). (E and F) Viral particles that exhibited gold beads labeling Rab11 in the viral envelope (arrows). (G) Viral particles that exhibited dual labeling with Rab11 (ultrasmall beads) and LC3B (10-nm beads). (H) Representative viral particles that did not exhibit labeling with a control antireovirus antibody. (I) Representative viral particles that exhibited gold beads labeling the VZV ORF41 capsid protein. (J) Enumeration of percentages of viral particles with at least one gold bead. Over 30 viral particles on grids in panels A to F and 112 particles on the grid in panel H were examined for gold beads. Each immunolabeling experiment was repeated twice. Error bars represent SD.

Under the secondary envelopment model of herpesvirus assembly, viral capsids migrate from the nucleus after deenvelopment and enter vesicles in the cytoplasm that are populated by viral envelope and tegument proteins as well as cellular proteins (41). The cytoplasmic vesicles carrying the viral particles are then transported to the outer cell membrane. Therefore, we postulated that the autophagy (LC3B) and endosomal (Rab11) proteins would be found on some purified viral particles if examined by immunoelectron microscopy (immuno-EM). We performed individual immuno-EM experiments with antibodies to gE, LC3B, and Rab11 applied to material from the viral fractions (Fig. 3) bound to TEM grids and then negatively stained with uranyl acetate. We have previously shown by immuno-EM that high-affinity MAb 3B3 attaches to the envelope of the VZV virion (15). Our present experiment confirmed our previous observation and furthermore showed that the anti-gE antibody attached to essentially all enveloped or partially enveloped viral particles (Fig. 5A and B); note the frequent presence of more than one gold bead on each viral particle.

Subsequently, we immunolabeled the viral fractions with LC3B and Rab11 antibodies. We were at a disadvantage because the binding affinities of the latter antibodies have not been characterized. Therefore, each of the following experiments was performed with at least two TEM grids per antibody. We also compared the sensitivities of secondary antibodies attached to ultrasmall gold beads (with silver enhancement) or 10-nm gold beads (without silver enhancement). Numerous images were taken, and representative examples are shown in Fig. 5. As shown in Fig. 5C and D, gold beads attached to LC3B were found on viral particles, and likewise, in Fig. 5E and F, gold beads attached to Rab11 were detected on particles. LC3B antibody attached to ∼30% of viral particles in the band of infectivity, while Rab11 beads were found on nearly 50% of particles (Fig. 5J). As is apparent in Fig. 5A to F, there were fewer gold beads/viral particle with either the anti-Rab11 antibody or anti-LC3 antibody than with MAb 3B3 against gE. However, we hesitate to draw broad conclusions because the antibodies to different proteins may have different affinities.

In order to evaluate whether both Rab11 and LC3B can be found on the same viral particle, we carried out a cryo-EM experiment whereby infected cells were embedded in gelatin, frozen, and then sectioned. This TEM method is preferred for greater antibody binding with a less-well-defined ultrastructure. Three viral particles were seen on the surface of an infected cell (Fig. 5G). Of importance, each particle exhibited multiple Rab11 beads (smaller gold bead) with fewer LC3B beads (larger gold bead). Furthermore, both Rab11 and LC3B beads, as would be expected, were found in the infected cell cytoplasm and on the infected cell plasma membrane.

For a negative-control experiment, we applied the antireovirus MAb (isotype IgG2a) along with secondary goat anti-mouse antibody (10-nm gold beads) to the grids and examined them by TEM. We enumerated a total of 112 viral particles; only 2 viral particles were observed with an adjacent gold bead. A representative micrograph is shown in Fig. 5H. An additional positive-control experiment was carried out with the MAb against the VZV ORF41 capsid protein. Note the presence of 5 gold beads on a viral particle (Fig. 5I, arrows). In summary, we found both vesicular components Rab11 and LC3B on purified viral particles in various amounts (Fig. 5J).

### Visualization of viral particles within cytoplasmic vesicles.

**FIG 6:**
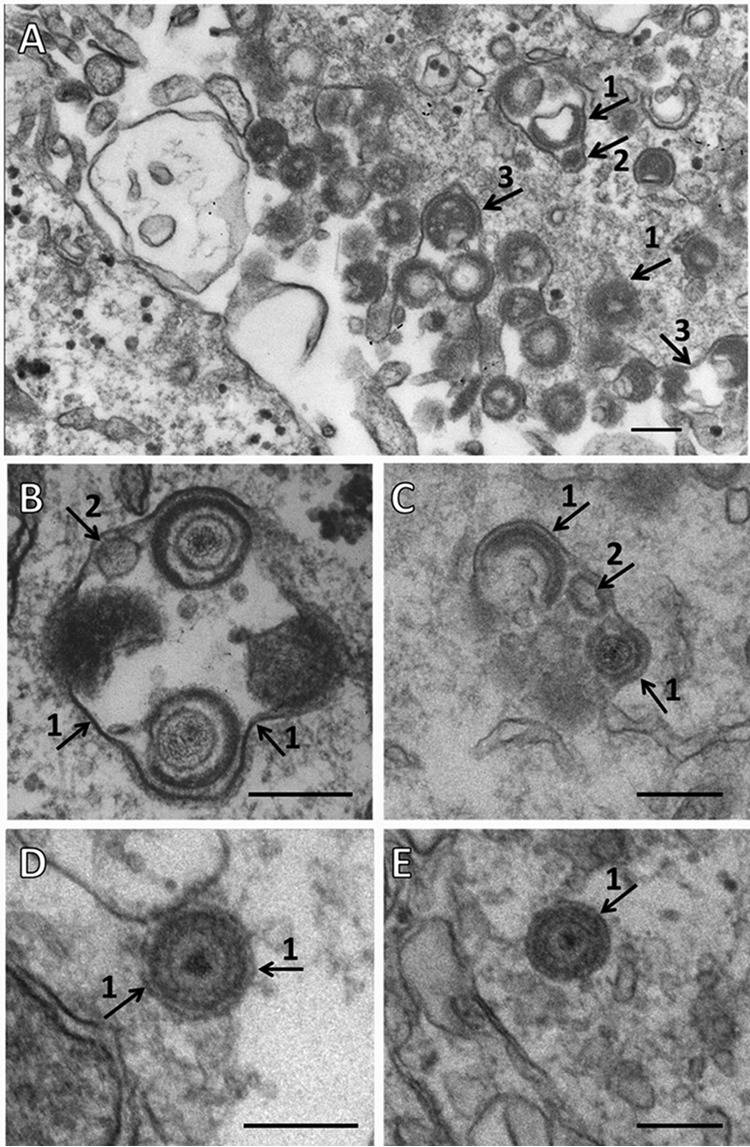
Visualization of vesicles carrying enveloped VZV particles. Over 100 electron micrographs were examined, and 5 were selected as representative examples. (A) Landscape view of a VZV-infected cell with numerous viral particles along the outer cell membrane. These particles are never released into the culture medium. This micrograph also shows one cytoplasmic vesicle containing two viral particles with a smaller spherical cargo and another smaller vesicle containing one viral particle. Single-walled vesicle membranes are designated by arrow 1, cargo within a vesicle is designated by arrow 2, and the outer cell membrane is designated by arrow 3. (B) Cytoplasmic vesicle containing two prototypical enveloped virions, two likely viral particles (not in same focal plane), and a smaller spherical cargo. (C) Cytoplasmic vacuole containing two viral particles and a smaller cargo. (D) Cytoplasmic vesicle containing one prototypical virion and no apparent cargo. (E) Another cytoplasmic vesicle containing one prototypical virion and no apparent cargo. Note that in panels D and E, the outer rim of the viral envelope and the single membrane of the surrounding vesicle should not be confused with a double-membraned autophagosome. Bar = 200 nm.

Because of the above-described observations about (i) copurification of LC3B and Rab11 proteins with the sedimented virus band (containing gE) as well as (ii) colocalization of the same two proteins with gE, we pursued further investigations by TEM. First, we wanted to confirm that viral particles were not found in typical double-membraned autophagosomes within VZV-infected monolayers (15, 16). As noted in an important HSV study of xenophagy, the presence of VZV particles in autophagosomes would infer a degradative pathway for the virus (42). Therefore, we reexamined >100 electron micrographs taken as part of completed but unreported projects. Again, all viral particles were observed in vesicles with a single bounding membrane within the cytoplasm of infected cells, especially near the outer cell membrane. A sampling of these micrographs is included in Fig. 6. We point out an important observation from the Seglen laboratory that amphisomes can be distinguished by TEM because they frequently contain small amounts of cargo that is transferred from their partial origin as an autophagosome (43). Note that vesicles containing 2 to 4 viral particles frequently had small spherical cargos that were <50 nm in diameter (Fig. 6A to C, arrow 2). Membranes around a single VZV particle were tightly wrapped (Fig. 6A to C, arrow 1); nevertheless, careful inspection of several of these vesicles at a higher magnification never revealed a double-walled autophagosome surrounding a viral particle (xenophagy). Two examples of a single virus within a single-membraned vesicle are illustrated in Fig. 6D and E. Finally, we note that this combination of a viral envelope surrounded by a tightly wrapped vesicle membrane can be mistaken for a double-walled membrane if not viewed at a higher magnification. Electron micrographs of uninfected monolayers were reported previously by our laboratory (16).

### VZV and nuclear envelope-derived autophagy.

**FIG 7:**
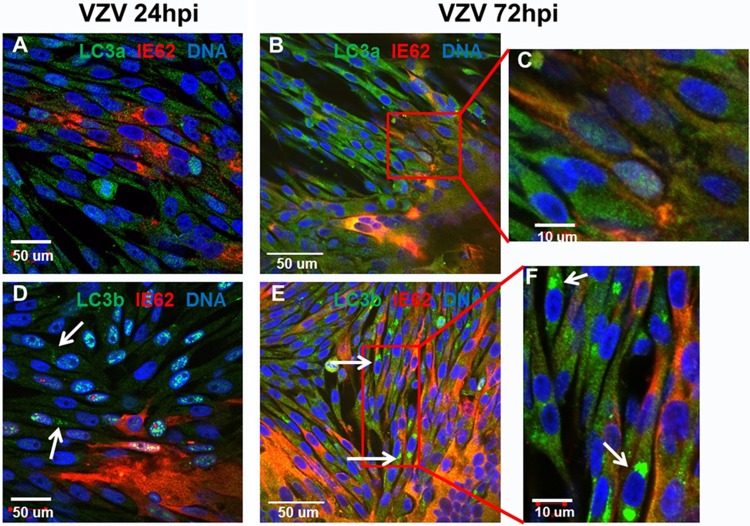
Failure to detect LC3A nuclear envelope-derived autophagy. Melanoma cells were infected and incubated for 24 h (early infection) (A and D) or 72 h (late infection) (B, C, E, and F). Cells were subsequently fixed, permeabilized, and then incubated with primary antibodies, including rabbit anti-LC3B (1:100), rabbit anti-LC3A (1:100), and murine anti-VZV ORF62 protein (MAb 5C6) (1:500) from our laboratory. Bars are shown for each image. LC3A staining failed to detect nuclear envelope autophagosome-like structures (A to C). LC3B staining identified conventional autophagosomes (white arrows in panels D to F).

Based on autophagy data in the HSV literature, we investigated whether an alternative source of LC3-II in VZV particles may be LC3A resident in the nuclear membrane. LC3A is localized primarily in the nuclear and perinuclear regions, while LC3B is found throughout the cytoplasm (44). All above-described experiments in this report were carried out with reagents against LC3B (see Materials and Methods). Nascent herpesviruses exit the nucleus and transit through the inner and outer nuclear membranes by a process of primary envelopment and deenvelopment of the capsid before trafficking to a virus assembly compartment in the cytoplasm for secondary envelopment of the capsid and exocytosis. In HSV-1-infected murine macrophage cells, investigators previously reported finding 4-layered structures that emanate from the nuclear membrane; these nuclear membranes were positive for the LC3A protein, and this process was called nuclear envelope-derived autophagy (45). Because of these reports, we performed similar experiments on VZV-infected monolayers, using the same anti-LC3A reagent as the one used by those HSV investigators (Fig. 7). For this experiment, we used MAb 5C6 against the VZV IE62 protein in order to ensure that we did not miss an early focus of VZV infection not detectable by anti-gE MAb. However, in VZV-infected cells, we were unable to detect comma-shaped 4-layered LC3A-positive structures originating in the nuclear membranes of the VZV-infected monolayers, at either 24 h postinfection (hpi) (Fig. 7A) or 72 hpi (Fig. 7B and C). Conventional LC3B-labeled autophagosomes were seen (Fig. 7D to F). We concluded that perinuclear autophagosome-like structures derived from LC3A were not found in VZV-infected cells and therefore did not participate in the transfer of LC3 protein to nascent VZV virions.

## DISCUSSION

This study provides insight into a previously unrecognized relationship between autophagy and the VZV infectious cycle. A proviral role for autophagy during herpesvirus infection was initially overlooked. The main explanation was the frequently cited pivotal report in 2007 that identified the HSV-1 neurovirulence protein ICP34.5 as an inhibitor of autophagy (46) The neurovirulence protein acquired its name because it facilitates HSV-1 replication in brain tissue; mutant HSV genomes lacking ICP34.5 are far less neurovirulent (47). Neurovirulence was correlated with the inhibition of autophagy by experiments demonstrating that ICP34.5 interacts with Beclin-1, thereby downregulating autophagy and enhancing HSV-1 replication in the brain. Of note for this report, the VZV genome, which is smaller than the HSV-1 genome (152 kbp), lacks a homolog of ICP34.5 (31). Furthermore, the VZV genome also lacks homologs of a second HSV-1 gene (ORF US11) as well as a cytomegalovirus gene, TRS1, both of which are associated with inhibition of autophagy (48, 49).

The induction of autophagy by VZV infection is easily observed in cultured cells by confocal laser scanning microscopy (7, 50). When the degree of VZV-induced autophagy was compared with those of typical inducers of autophagy such as serum starvation or trehalose, the increase in autophagy as gauged by the enumeration of autophagosomes was similar (9, 51). Furthermore, treatment of infected cells with trehalose markedly increased both the total amount of viral glycoprotein as well as the glycan processing of viral glycoprotein found in the virion band in density gradients. Taken together, these results suggested that autophagy was involved in secondary viral envelopment; in turn, secondary envelopment is closely linked to exocytosis of the enveloped particle. To investigate this hypothesis, we again purified virus by optimal sedimentation conditions that are required for the preservation of infectious virus (26, 27). Using density-viscosity gradients to prepare highly purified virus, we documented the presence of autophagy and endosomal proteins in the virion band. We relied upon the gE protein as a marker for virions because the linear epitope for anti-gE MAb 3B3 is defined, and the binding affinity nearly equals that of the FLAG antibody (20, 52).

These results suggested a convergence between the autophagy pathway and the virus assembly/egress pathway. The fact that both LC3-II and Rab11 copurify with VZV virions is an important observation, because this finding indicates that there is not a specific mistrafficking or redirecting of a single cellular component into the viral envelopment pathway. This point is relevant because Epstein-Barr virus (EBV) researchers recently reported finding LC3 in the EBV virion, but further characterization of other autophagy proteins was not performed (53). Little is known about the VZV assembly compartment other than that it appears to be located near the *trans*-Golgi network and/or recycling endosome (15, 54). Of interest, 4 major viral glycoproteins harbor endocytosis sequences in their C tails (18, 55). For the predominant VZV gE protein, we have shown that the glycoprotein traffics from the Golgi membrane to the outer cell membrane, where it is internalized in clathrin-coated vesicles together with the transferrin receptor (56, 57). VZV gE is sometimes called the navigator protein, because it directs trafficking of the VZV glycoprotein complexes, presumably to the recycling endosome (38, 58).

**FIG 8:**
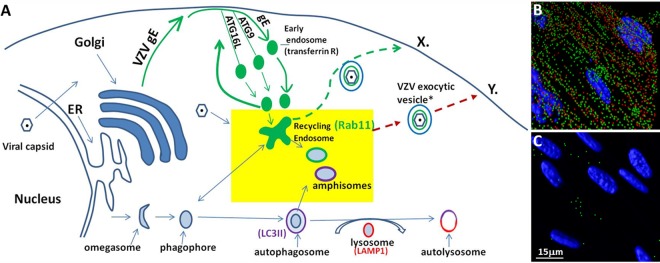
Trafficking pathways shared by the major VZV glycoprotein gE with autophagy proteins. (A) Schematic summary of trafficking pathways. VZV gE is known to traffic from the ER/Golgi membrane to the outer cell membrane, where it undergoes clathrin-mediated endocytosis and transits to a recycling endosome along with the transferrin receptor. The VZV gE pathway overlaps the endocytosis pathway of two autophagy proteins, Atg9 and Atg16L, both of which transit to the recycling endosome *en route* to the autophagosome pathway. The site of the VZV assembly compartment has not been clearly defined but is presumed to be in proximity to the recycling endosome. The yellow rectangle highlights the proposed VZV assembly area, where vesicles (Rab11^+^) enclosing newly formed VZV virions (gE^+^) could fuse with LC3-II-positive vesicles. The two different vesicles represent a heterogeneous population of amphisomes or amphisome-like organelles that carry newly enveloped virions (64). The red dashed arrow shows the proposed exocytosis pathway of these single-membraned vesicles (Y at the cell surface). The green dashed arrow shows a conventional route of exocytosis directly from the recycling endosome (X at the cell surface). LAMP1, lysosome-associated membrane protein 1. (B) Imaris rendering of an infected monolayer. Viral highways are present near the cell surface. Red, VZV gE; green, Rab11. (C) Imaris rendering of an uninfected monolayer. Note the absence of red puncta. The bar represents both panels B and C.

We prepared a schema to illustrate the known trafficking patterns of VZV gE within the virus life cycle, as documented by two VZV laboratories (38, 59, 60). Of importance, two Atg proteins, Atg9 and Atg16L1, traffic in a similar endocytosis pathway with the transferrin receptor, as they are routed from the outer cell membrane to a recycling endosome (61, 62). Rab11 in turn traffics in recycling compartments to the outer cell membrane (63). When we superimposed the trafficking patterns of the proteins, the similarities overall were striking (Fig. 8). As mentioned above, the gE protein is known to traffic to the outer cell membrane after synthesis and processing in the endoplasmic reticulum (ER)/Golgi membrane. The glycoprotein can travel by itself, probably as a dimer or trimer, or it can traffic together with its partner, gI, another type 1 glycoprotein (18). At the outer cell membrane, they are internalized by clathrin-mediated endocytosis and then traffic in the endosomal pathway. Although very little research has been carried out on the VZV assembly compartment, we presume, based on other herpesvirus data, that this compartment is located in proximity to the recycling endosome (41). We postulate that the gE glycoprotein traffics to the virus assembly compartment, where the viral particles undergo secondary envelopment. Some nascent virions are transported in a recycling-derived vesicle (Rab11^+^) that fuses with an autophagosome (LC3^+^) to form a single-walled vesicle containing both Rab11 and LC3-II; these vesicles in turn traffic to the outer cell membrane. Based on the data in this report, we therefore propose that there are at least 2 pathways by which virus can egress from the infected cell. The first pathway (labeled X in Fig. 8A) is the conventional pathway that is cited in the literature (41). The second pathway (labeled Y in Fig. 8A) is the pathway with LC3-positive vesicles. These vesicles in turn traffic to the outer cell membrane, where they form topographically distinctive viral highways (Fig. 8B) that are not present on uninfected monolayers (Fig. 8C).

In a recent commentary (64), amphisomes were described as a heterogeneous population of single-walled compartments where parts of the autophagy and endosomal machineries colocalize. Based on our data about the colocalization of LC3-II and Rab11 with purified enveloped virus (gE positive), we postulate that some enveloped VZV particles are likely housed in this heterogeneous population of LC3-positive single-walled compartments (Fig. 8). In addition to the protein markers, the vesicles also contained features seen by TEM that were commensurate with the description of an amphisome by the Seglen laboratory (43). During our extensive observations by TEM, we noted that some vesicles harbor several particles, while other vesicles contain only one or two viral particles (15, 16). We postulate that vesicles (LC3/Rab11^+^) containing only 1 or 2 particles remain intact (or mainly intact) throughout the purification process and therefore are detected by LC3/Rab11 immunoblotting in the virus band. The only other single-walled compartment with the LC3-II protein in its outer membrane is the autolysosome. Since the autolysosome transitions into a lysosome and does not traffic to the outer cell membrane, we eliminated the autolysosome as a candidate trafficking vesicle for viruses.

In a recent comprehensive review of secondary envelopment of alphaherpesviruses, the autophagy pathway was not recognized as a contributor (41). Therefore, this new VZV observation alters the alphaherpesvirus paradigm. In a previous investigation of the trafficking of wild-type HSV strain 17 and a mutant construct, HSVΔ34.5, mutant viral particles were observed within autophagosomes, while wild-type HSV-1 was found only in single-membraned cytoplasmic vesicles (42). Those investigators concluded that the absence of the ICP34.5 gene facilitated the transit of HSV particles into an autophagosome, which would transition into a lysosome (Fig. 8), while the presence of the ICP34.5 gene allowed HSV particles to traffic in single-membraned vesicles to the outer cell membrane. Since the VZV genome lacks the ICP34.5 gene, the absence of VZV particles in autophagosomes suggests that the ICP34.5 protein by itself does not determine whether HSV particles transit within double-membraned cytoplasmic vesicles (presumably to their degradation in lysosomes) instead of single-membraned vesicles (presumably to their release on the cell surface). Investigators studying two gammaherpesviruses, Epstein-Barr virus and Kaposi's sarcoma-associated herpesvirus, proposed recently, based on TEM data, that both gammaherpesviruses are transported in autophagosomes following viral assembly (65, 66). Thus, even between subfamilies of herpesviruses (alpha- versus gammaherpesviruses), there are small but remarkable differences in transport vesicles within the cytoplasm. Further clarification of these differences will require the development of techniques to isolate and characterize exocytosis vesicles containing virions.

Some RNA viruses exploit endosomal and autophagy pathways for their replication and release from cells (67). Poliovirus relies on autophagosomes, or double-membraned vesicles, for nonlytic viral spread (68). Hepatitis C virus hijacks the autophagy machinery to improve its replication, and activation of autophagy has been shown to play a crucial role in the release of viral particles via undefined mechanisms (69). Furthermore, serotypes of dengue virus have been shown to use components of the autophagy pathway as a platform for RNA replication (70), while hepatitis A virus exploits the endosomal sorting pathways to exit cells (71). Recently, two RNA viruses have been shown to use Rab11 endosomal components to exit cells (72, 73). The latter Rab11 observation is striking because it implies a much closer concordance in exit strategies among some RNA viruses and DNA viruses, such as VZV, than had been appreciated previously.

## Supplementary Material

Supplemental material
