# Supplementary material for: Exocytosis of Varicella-Zoster Virus Virions Involves a Convergence of Endosomal and Autophagy Pathways
Source: J Virol. 2016 Sep 12;90(19):8673–85. doi: 10.1128/JVI.00915-16 (PMC5021422; doi:10.1128/JVI.00915-16)
Supplement: Supplemental material [file supp_90_19_8673__index.html]

Exocytosis of Varicella-Zoster Virus Virions Involves a Convergence of Endosomal and Autophagy Pathways — Supplemental material 

# Exocytosis of Varicella-Zoster Virus Virions Involves a Convergence of Endosomal and Autophagy Pathways

## Supplemental material

- Supplemental file 1 -

  Legend to video S1

  PDF, 8.5K
- Supplemental file 2 -

  Video S1 (Colocalization of Rab11 and VZV gE in puncta after rendering by Imaris software.)

  AVI, 6.5M
