# Supplementary material for: Exocytosis of Varicella-Zoster Virus Virions Involves a Convergence of Endosomal and Autophagy Pathways
Source: J Virol. 2016 Sep 12;90(19):8673–85. doi: 10.1128/JVI.00915-16 (PMC5021422; doi:10.1128/JVI.00915-16)
Supplement: Supplemental material [file JVI.00915-16_zjv999181974so1.pdf]

Supplemental video S1 legend

Colocalization of Rab11 and VZV gE in puncta after rendering by Imaris software. VZV-infected MRC-5 monolayers were fixed and labeled with anti-Rab11 antibody, anti-VZV gE antibody and Hoescht 33342 DNA stain. Numerous confocal images from z-stacks were generated into 3D animations using the surface and spot tools in Imaris software. The spot detection was carried out after adjustment of the spot function to 1000 nm. Cell nuclei are colored blue, Rab11 labeled puncta are portrayed as green spheres and gE labeled puncta are portrayed as red spheres; colocalization of gE and Rab11 is illustrated by spheres that are half red/half green. The 3D animation in the z plane illustrates puncta above the nuclei while leaving the nuclei intact.
